# A Case of Low-Grade Oncocytic Tumor/Chromophobe Renal Cell Carcinoma (Oncocytic Variant) of the Kidney

**DOI:** 10.1155/2021/6684777

**Published:** 2021-02-18

**Authors:** Noriyoshi Ishikawa, Nao Kimura, Toshio Yoshida, Ichiro Yoshimura, Ken Nakahara, Toyonori Tsuzuki, Osamu Tokunaga

**Affiliations:** ^1^Department of Pathology, Shonan Fujisawa Tokushukai Hospital, 1-5-1 Tsujido-kandai, Fujisawa, Kanagawa 251-0041, Japan; ^2^Department of Urology, Shonan Fujisawa Tokushukai Hospital, Japan; ^3^Department of Surgical Pathology, Aichi Medical University, School of Medicine, Japan

## Abstract

The oncocytic variant of chromophobe renal cell carcinoma (oChRCC) and low-grade oncocytic tumor (LOT) is introduced as new renal disease entity. Both of these tumors are low-grade malignancies consisting of cells with eosinophilic cytoplasm. Distinguishing between eosinophilic variant of chromophobe renal cell carcinoma (eCRCC) and oncocytoma is often a diagnostic challenge in routine surgical pathology. However, oChRCC and LOT might be independent disease entities that might not fit completely into any of these categories. Histologically, these tumors have greater morphological similarity with oncocytoma than with ChRCC. However, immunohistochemically, they exhibit diffuse and dense positivity for CK7 and are negative for CD117. In the present case, we initially had difficulty distinguishing among oncocytoma, eCRCC, and type 2 papillary renal cell carcinoma (2-pRCC). However, after learning about new disease entities such as oChRCC and LOT, we were able to diagnose this tumor.

## 1. Introduction

There are many types of oncocytic/eosinophilic renal tumors, which often present difficulties in diagnosis [1]. Among the well-defined entities, a definitive diagnosis may not be obtained even after using immunostaining techniques. At the same time, many new disease entities have been reported in recent years.

Well-defined oncocytic/eosinophilic renal tumor entities that need to be distinguished from one another include oncocytoma, eosinophilic variant of chromophobe renal cell carcinoma (eCRCC), clear cell RCC, type 2 papillary renal cell carcinoma (2-pRCC), and epithelioid AML [2]. Among these, it is difficult to distinguish between oncocytoma and eCRCC [1].

On immunostaining, eCRCC generally exhibits diffuse positivity for CK7, whereas oncocytoma is negative or only partially positive [1]. Thus, these two tumors can be smoothly differentiated in many cases. However, cases are often experienced wherein these tumors cannot be differentiated even with various immunostaining techniques. Diffuse and dense CK7 positivity has been observed even in tumors that were morphologically similar to oncocytoma [3–5].

Moreover, new disease entities have been discovered in recent years. Differentiation diagnosis is important for oncocytic/eosinophilic renal tumors [6] including succinate dehydrogenase- (SDH-) deficient renal cell carcinoma [7], thyroid-like follicular RCC [8], ALK-rearrangement RCC [9], high-grade oncocytic renal tumor [10], LOT [4, 5], oChRCC [3], and eosinophilic solid and cystic renal cell carcinomas [11]. Among these, oChRCC and LOT are tumors that are similar to oncocytoma but are characterized by diffuse and dense immunoreactivity for CK7 [2, 4, 5].

## 2. Case Report

A left renal tumor was observed on abdominal computed tomography (CT) during examination in a 78-year-old man being treated for atrial fibrillation. Subsequently, the patient was referred to our hospital.

**Figure 1:**
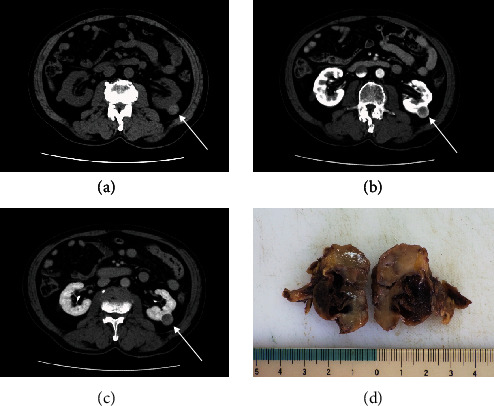
(a) Plain CT: tumor showed slightly dense signal (white arrow). (b) Contrast-enhanced CT: well-defined solid mass was enhanced around the tumor in the early phase (white arrow). (c) Contrast-enhanced CT: tumor was washed out in the late phase (white arrow). (d) Cut surface of the tumor. cystic area was observed inside the tumor.

Abdominal CT showed a mass with a maximum diameter of 2.2 cm, protruding to the capsule side of the kidney. The mass showed faint signal intensity on plain CT (Figure 1(a)) and intense early enhancement around tumor (Figure 1(b)) and washout in the late phase on contrast-enhanced CT (Figure 1(c)). The cystic area was also observed inside the mass (Figure 1(b)). Partial left nephrectomy was performed for this tumor.

**Figure 2:**
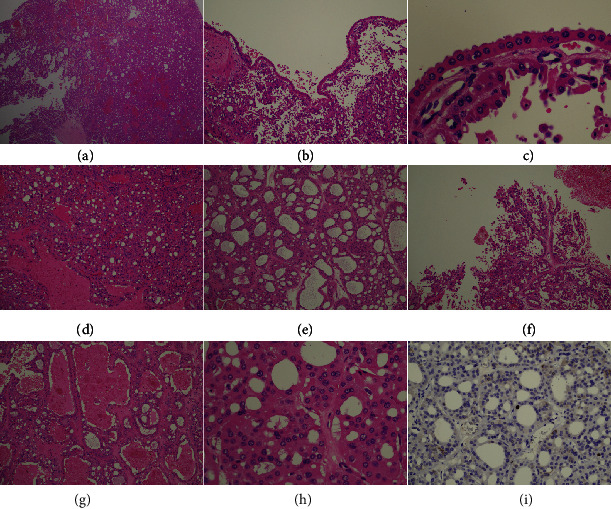
(a) Solid area of the tumor. (b) Cystic area of the tumor. (c) Lining cells of cyst are composed of eosinophilic cells. (d) Tumor cells are proliferative with microcystic component. (e) Tumor cells are predominantly proliferative with tubular pattern with dilated tubules. (f) Small amount of papillary pattern in the tumor. (g) Many dilated tubules were seen in the tumor. (h) Enlargement of nuclear but mild atypia of eosinophilic tumor cells. (i) Tumor cells of Mib-1 index are very low.

**Figure 3:**
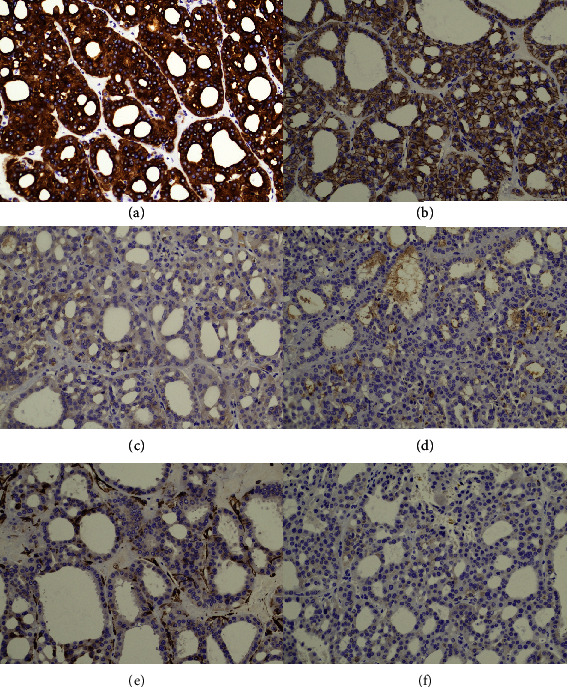
(a) CK7: diffuse and densely positive. (b) E-cadherin: diffusely positive. (c) c-kit. (d) CD10. (e) Vimentin. (f) Alpha-methylacyl-CoA racemase.

A cyst was observed at the center of the mass on the cut surface, and a solid dark brown-colored area was observed around the cyst (Figure 1(d)). Histologically, tumor cells with oncocytic/eosinophilic cytoplasm had proliferated in both the solid part (Figure 2(a)) and the lining of the cyst (Figures 2(b) and 2(c)). The tumor cells had proliferated predominantly in microcystic to a tubular pattern (Figure 2(d)) with dilated tubules (Figure 2(e)) and showed a focal papillary pattern (Figure 2(f)). Some dilated tubules contained blood components (Figure 2(g)). Although nuclear enlargement of tumor cells was observed, nuclear atypia was weak (Figure 2(h)) and the MIB-1 index was approximately 1% (Figure 2(i)). A fibrous capsule of the tumor was inconspicuous, but no progression to the surrounding areas was observed. There were no edematous stromal areas in the tumor. The tumor stage was pT1a, and no vascular invasion was observed. On immunostaining, tumor cells exhibited diffuse and dense CK7 positivity (Figure 3(a)) and diffuse E-cadherin positivity (Figure 3(b)). In contrast, tumor cells were negative for c-kit (Figure 3(c)), CD10 (Figure 3(d)), vimentin (Figure 3(e)), and alpha-methylacyl-CoA racemase (Figure 3(f)). Based on these findings, we diagnosed the tumor as LOT/oChRCC, although the morphology was somewhat different from previously reported cases.

## 3. Discussion

The classification of renal tumors with oncocytic/eosinophilic cytoplasm has been rapidly organized in recent years. Particularly, tumors that have emerged as new entities in recent years include SDH-deficient RCC [7], thyroid-like follicular RCC [8], ALK-rearrangement RCC [9], high-grade oncocytic renal tumor [10], oChRCC [3], LOT [4, 5], and eosinophilic solid and cystic RCC [11]. Oncocytoma, eCRCC, clear cell RCC, and 2-pRCC are well-defined entities that are also listed as differential diagnoses [2].

In routine surgical pathology, the distinction between eChRCC and oncocytoma is a frequent dilemma. The former is a low-grade malignant tumor, and the latter is a benign tumor. Barring this distinction, there is not much difference in the prognosis of these two diseases. However, if the tumor is diagnosed as malignant, life insurance payment will be involved. In addition to the morphological findings in hematoxylin and eosin (HE) staining, immunostaining is a very useful tool for differentiating between these tumors. Immunostaining for CK7 is the most useful method wherein eCRCC often exhibits diffuse positivity, but oncocytoma is only partially positive [1].

However, Kuroda et al. reported five cases of oncocytoma-like tumors (termed oChRCC) that were diffusely and densely positive for CK7 [3]. This tumor proliferates predominantly in a tubular pattern and sometimes shows proliferation in a solid pattern [3]. In addition, the nuclei of the tumor cells are centrally located and do not exhibit the perinuclear halo observed in ChRCC [3]. It also has no myxedema-like stroma [3].

A few years later, Trpkov et al. reported 28 similar tumors that were termed LOTs [4]. LOT is more common in women and is often discovered at an early stage [4]. The prognosis was good in all reported cases, and no deaths were reported [4]. LOT is similar to oChRCC reported by Kuroda et al. In both the cases, the tumor boundary was clear and there was no capsule formation [4]. However, oChRCC frequently exhibits myxedema-like stroma unlike LOT [3, 4]. Additionally, the tumors reported by Kuroda et al. were proliferative with predominantly tubular growth pattern [3], while the tumors reported by Trpkov et al. were predominantly solid and often exhibited nested patterns [4]. The tumors reported by Kuroda et al. did not have any perinuclear halo [3], but the tumors reported by Trpkov et al. often exhibited perinuclear halo [4]. Although the test methods for chromosomal abnormalities were different in these two reports, most of the tumors reported by Kuroda et al. had deletions of chromosomes 7, 10, 13, 17, and 21 [3]. On the other hand, many tumors among those reported by Trpkov et al. exhibited 19p33.3 and 1p36.33 deletions [4]. Guo et al. reported eight tumors that exhibited features consistent with the tumor entities described by Trpkov et al. [5]. The cases reported by Guo et al. were morphologically similar to the cases reported by Trpkov et al., and many of them had features consistent with LOT [5].

The tumor in the present case had greater similarity with oChRCC than with LOT due to features such as predominantly tubular growth pattern instead of solid pattern, no myxedema-like stroma, and no perinuclear halo. In addition, the tumor in the present case had some differences in morphological features such as the presence of a cyst within the tumor and focal papillary structure when compared with previously reported tumors.

Based on the immunostaining results, the tumors reported by Kuroda et al. and Trpkov et al. are currently considered closely related renal tumors. Hence, we diagnosed the tumor in the present case as oChRCC or LOT. The relationship between these two tumors will be clarified after the accumulation of more information from future cases. It might be preferable to use the term oncocytic variant of chromophobe renal cell tumor (oChRCT) in place of oChRCC.

Without the knowledge about these new disease entities, the tumor might have been misdiagnosed as oncocytoma based on the tumor morphology in HE staining. However, due to diffuse and dense positivity for CK7 on immunostaining, eChRCC was also a potential differential diagnosis. Hence, we might have diagnosed the tumor as a hybrid tumor or unclassified renal cell carcinoma, which would be annoying to clinicians. The reports describing these tumors are still small in number. However, irrespective of the diagnosis (oChRCC or LOT), a good prognosis is expected even after follow-up alone due to the indolent behavior of both tumors.

Some oncocytic/eosinophilic renal tumors still do not fit into the currently known diagnostic categories, and research using genetic testing is still in progress. Hence, we believe that the number of reported tumors will increase in the future and pathologists should always keep an eye on the new information. The classification of oncocytic/eosinophilic tumors might be organized in the near future. The category of the tumor in the present case was unclear. However, we believed that it was important to report the detailed histology of the tumor.

## Data Availability

All supporting data (macroscopic and microscopic imaging) are included in the manuscript.
